# Behavioral and psychological symptoms of dementia (BPSD) in Oldest Old Nigerians: A 20‐year Follow‐up of Indianapolis‐Ibadan Dementia Project

**DOI:** 10.1002/alz70857_096605

**Published:** 2025-12-24

**Authors:** Olufisayo Oluyinka Elugbadebo, Olusegun Baiyewu, Hephzibah Oyedapo‐Ishola, Rufus O. Akinyemi, Hugh C Hendrie, Akin Ojagbemi, Temitope Hannah Farombi, Adesola Ogunniyi, Kathleen A. Lane, Sujuan Gao, Farid Rajabli, Michael L. Cuccaro, Pedro R Mena, Jeffery M Vance, Margaret Pericak‐Vance

**Affiliations:** ^1^ College of Medicine, University of Ibadan, Ibadan, Nigeria; ^2^ University College Hospital, Ibadan, Nigeria; ^3^ University College Hospital, Ibadan, Oyo State, Nigeria; ^4^ University College Hospital, Ibadan, Oyo, Nigeria; ^5^ College of Medicine, University of Ibadan, Ibadan, Oyo State, Nigeria; ^6^ Indiana University School of Medicine, Indianapolis, IN, USA; ^7^ College of Medicine, University of Ibadan, Ibadan, Oyo, Nigeria; ^8^ Institute for Advanced Medical Research and Training, College of Medicine, University of Ibadan, Ibadan, Nigeria; ^9^ Indiana University, Indianapolis, IN, USA; ^10^ School of Medicine, Indiana University‐Purdue University at Indianapolis, Indianapolis, IN, USA; ^11^ John P. Hussman Institute for Human Genomics, University of Miami Miller School of Medicine, Miami, FL, USA; ^12^ University of Miami Miller School of Medicine, Miami, FL, USA

## Abstract

**Background:**

Behavioral and Psychological Symptoms of Dementia (BPSD) alongside cognitive decline and functional impairment are a part of the symptoms complex of dementia. BPSD is responsible for the enormous amount of caregiver distress and results in an increase in hospitalization, institutional placement, and mortality. Few studies have examined the BPSD among oldest‐old with dementia and cognitive impairment in the developing world. Thus, this study appraised BPSD among the surviving participants from our original Indianapolis‐Ibadan Dementia Study and assigned diagnosis.

**Methods:**

Participants were the survivors of the Ibadan arm of the Indianapolis‐Ibadan Dementia Project who were enrolled in 1992 or 2001 and re‐examined between 2021 and 2022. Neuropsychological assessment was with Consortium to Establish a Registry for Alzheimer Disease (CERAD) Neuropsychological battery. Diagnosis was based on DSM IV TR and National Institute of Aging criteria and classified as normal cognition, mild cognitive impairment (MCI), and dementia. Subjects were further assessed with the use of Neuropsychiatric Inventory Questionnaire (NPI‐Q) for the prevalence and severity of BPSD, and caregiver distress caused by those symptoms by each subject diagnostic group.

**Results:**

From our study, 135 survivors were re‐examined and diagnosed in 2021/2022 with a mean age of 92.5 years. Sixty‐two participants (46.1%) had normal cognition, 44 (32.4%) had MCI and 29 (21.5%) had dementia. Depression was the most frequent BPSD among the MCI (27.3%) and dementia (34.5%) groups compared to the normal cognition group (14.5%). The dementia group exhibited the highest BPSD severity scores (4.20 ± 4.49), with appetite change being the most severe symptom across all groups. The highest distress scores were found among the caregivers of participants in the dementia group (2.21 ± 4.14) with appetite change and depression being the most distressful symptoms.

**Conclusions:**

The prevalence of depression among the participants and the resulting distress on their caregivers were frequent across all diagnostic groups in this study. These findings highlight the crucial need for interventions tailored to mitigate depression and other symptoms among the oldest‐old population living with cognitive impairments in developing countries, as well as address the resultant distress experienced by their caregivers.

